# Conserved roles of chromatin remodellers in cohesin loading onto chromatin

**DOI:** 10.1007/s00294-020-01075-x

**Published:** 2020-04-10

**Authors:** Sofía Muñoz, Francesca Passarelli, Frank Uhlmann

**Affiliations:** Chromosome Segregation Laboratory, The Francis Crick Institute, London, UK

**Keywords:** Cohesin, Chromatin remodellers, RSC, Scc2–Scc4, Cornelia de lange syndrome, Coffin–Siris syndrome

## Abstract

Cohesin is a conserved, ring-shaped protein complex that topologically entraps DNA. This ability makes this member of the structural maintenance of chromosomes (SMC) complex family a central hub of chromosome dynamics regulation. Besides its essential role in sister chromatid cohesion, cohesin shapes the interphase chromatin domain architecture and plays important roles in transcriptional regulation and DNA repair. Cohesin is loaded onto chromosomes at centromeres, at the promoters of highly expressed genes, as well as at DNA replication forks and sites of DNA damage. However, the features that determine these binding sites are still incompletely understood. We recently described a role of the budding yeast RSC chromatin remodeler in cohesin loading onto chromosomes. RSC has a dual function, both as a physical chromatin receptor of the Scc2/Scc4 cohesin loader complex, as well as by providing a nucleosome-free template for cohesin loading. Here, we show that the role of RSC in sister chromatid cohesion is conserved in fission yeast. We discuss what is known about the broader conservation of the contribution of chromatin remodelers to cohesin loading onto chromatin.

## Introduction

Cohesin is a central regulator of chromosome architecture, performing prominent roles in sister chromatid cohesion, genome organization, transcriptional regulation and DNA repair. This is accomplished due to its ability of trapping one or more molecules of DNA inside its ring-shaped structure (Litwin and Wysocki 2018; Nasmyth and Haering 2009; Peters and Nishiyama 2012; Villa-Hernandez and Bermejo 2018). Cohesin loading onto chromosomes requires a specialised cohesin loader complex, comprised of the Scc2 and Scc4 subunits (Ciosk et al. 2000; Gillespie and Hirano 2004). In vitro, Scc2–Scc4 loads cohesin in a DNA sequence independent manner (Murayama and Uhlmann 2014), whereas in vivo, cohesin is loaded at specific chromosomal locations. The chromosomal loading sites of the cohesin complex at centromeres and promoters of certain highly transcribed genes have been known for some time (Kagey et al. 2010; Lopez-Serra et al. 2014; Petela et al. 2018; Zuin et al. 2014). However, the features that define these cohesin loading sites are incompletely understood.

In the budding yeast *Saccharomyces cerevisiae*, the ‘Remodels the Structure of Chromatin’ (RSC) chromatin-remodelling complex co-occupies the genomic cohesin loader locations and is necessary for the cohesin loader recruitment to those sites (Lopez-Serra et al. 2014). RSC is a member of the SWI/SNF (switch/sucrose non-fermentable) family of ATP-dependent chromatin remodellers that utilise ATP hydrolysis to move DNA along the histone octamer. RSC action causes nucleosome eviction and the widening of nucleosome-free regions that provide DNA accessible to the transcription initiation machinery and to additional factors involved in other DNA metabolism processes (Clapier et al. 2017). We recently described two separate roles of RSC in cohesin loading onto chromatin. On the one hand, RSC acts as a chromatin receptor that recruits Scc2–Scc4 by a direct protein interaction, independently of chromatin remodelling. On the other hand, RSC’s chromatin-remodelling activity is required to generate a nucleosome-free region that is the substrate for cohesin loading (Muñoz et al. 2019).

Mutations in NIPBL, the human ortholog of the Scc2 cohesin loader subunit, are the cause of Cornelia de Lange Syndrome (CdLS), a hereditary disorder whose clinical features are thought to be caused by the misregulation of gene expression programs during development (Krantz et al. 2004). CdLS shows overlapping clinical features with Coffin–Siris syndrome (Fryns 1986), which is caused by mutations in various subunits of the human SWI/SNF chromatin-remodelling complexes (Santen et al. 2012; Tsurusaki et al. 2014) suggesting the existence of a functional link between the human cohesin loader and RSC orthologs with important clinical implications. Another chromatin remodeller family, ISWI (imitation switch), has also been implicated in cohesin loading onto chromosomes in human cells (Hakimi et al. 2002), opening the possibility that additional or different chromatin remodeller classes contribute as chromatin receptors during cohesin loading.

## A role of RSC in sister chromatid cohesion in the fission yeast *Schizosaccharomyces pombe*

In budding yeast, inactivation of RSC using a thermosensitive allele of its ATPase catalytical subunit, Sth1, *sth1-3*, or by depleting Sth1 using an auxin-inducible degron, leads to cohesion defects. In contrast, sister chromatid cohesion remains intact upon the individual depletion of catalytical subunits of any of the other budding yeast chromatin remodellers (Huang et al. 2004; Lopez-Serra et al. 2014; Muñoz et al. 2019). To understand the role of different chromatin remodeller families in cohesin loading onto chromatin across evolution, we extended our investigation to another model eukaryote, the fission yeast *Schizosaccharomyces pombe*. This organism shares many aspects of chromatin organization with higher eukaryotes and, interestingly, it utilises a distinctly different set of chromatin remodellers compared to those in budding yeast. Chromatin remodellers can be classified into four families: imitation switch (ISWI), chromodomain helicase DNA-binding (CHD), switch/sucrose non-fermentable (SWI/SNF) and INO80, according to the domain architecture of their catalytic ATPases (Clapier et al. 2017). *S. cerevisiae* mainly relies on ISWI family remodellers for chromatin assembly and owns three members of this family: Isw1a, Isw1b, and Isw2. There is only one CHD family member, the single subunit Chd1 remodeller. In comparison, *S. pombe* lacks any ISWI family member, while it expresses three CHD complexes, Hrp1, Hrp3, and Mit1. The latter in turn is a representative of the Mi-2/NuRD subcategory of CHD complexes that is missing in budding yeast. Both yeasts possess two members of the SWI/SNF family, whose catalytical subunits are Sth1 and Snf2 in *S. cerevisiae* and Snf21 and Snf22 in *S. pombe*. Similarly to RSC inactivation in budding yeast, *S. pombe* Snf21 depletion leads to shrinkage of nucleosome-depleted regions (Yague-Sanz et al. 2017). There are also two members of the INO80 family in both yeasts, named Ino80 and Swr1 in both species.

**Fig. 1:**
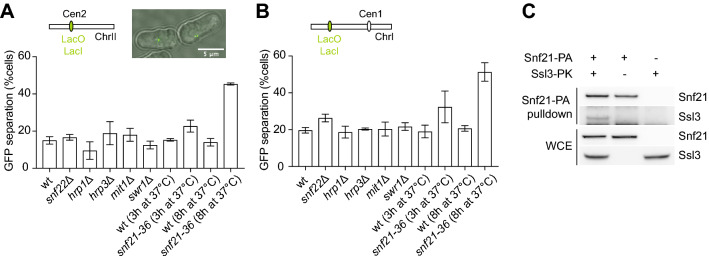
A role of RSC in sister chromatid cohesion is conserved in *S. pombe* **a** Sister chromatid cohesion at the centromere of chromosome II was scored in asynchronously growing cells of the indicated genotypes. Means and SD of three independent experiments are shown. An example of cells showing one or two GFP foci is shown. **b** As in **a**, but monitoring a GFP-marked locus on the long left arm of chromosome I. **c** Cell extracts were prepared and protein A-tagged Snf21 (Snf21-PA) was precipitated. Co-precipitation of Ssl3, fused to a Pk epitope tag, was analyzed by immunoblotting

To assess the contribution of chromatin remodellers to sister chromatid cohesion in fission yeast, we individually deleted non-essential catalytical subunits of *S. pombe* chromatin remodellers. We did this in a strain background harbouring an array of Lac operators next to the centromere of chromosome II and expressing a GFP-LacI repressor fusion protein to visualize this locus (Garcia et al. 2002). Like in budding yeast, the RSC remodeller ATPase ortholog Snf21 is an essential gene in fission yeast. We therefore used its thermosensitive mutant *snf21-36* that we inactivated at its restrictive temperature (Yamada et al. 2008). The status of sister chromatid cohesion was then assessed in asynchronously growing fission yeast cultures, in which the majority of cells are in the G2 phase of the cell cycle. A single GFP dot indicates efficient sister chromatid cohesion at centromere II in these G2 cells, while defective sister chromatid cohesion results in splitting of the GFP dot into two (Fig. 1a). A certain background fraction of cells with 2 GFP dots is expected to be visible in all cultures, which includes cells in the process of chromosome segregation during cell division. Deletion of *snf22*, *hrp1*, *hrp3*, *mit1* or *swr1* did not cause a discernible increase of cells with 2 GFP dots. In contrast, RSC complex inactivation using the *snf21-36* allele led to a marked sister chromatid cohesion defect (Fig. 1a). The defect became apparent 3 h following the shift to the restrictive temperature and was even more pronounced after 8 h. As an additional approach, we monitored sister chromatid cohesion along chromosome arms using Lac operators integrated at position 1.95 Mb in the middle of the chromosome I long left arm (Petrova et al. 2013). Similarly to what we observed at centromere II, cohesion along the chromosome I arm was compromised following inactivation of Snf21, but not in the absence of any of the other chromatin remodellers (Fig. 1b). These experiments suggest a conserved role of the RSC chromatin remodeller in sister chromatid cohesion, both at centromeres and along chromosome arms.

In addition to providing nucleosome-free DNA as a substrate for the cohesin loading reaction, RSC also acts as a chromatin receptor for the budding yeast cohesin loading complex via a direct protein–protein interaction (Muñoz et al. 2019). We, therefore, asked if this feature was also conserved in *S. pombe*. For that purpose, we performed a pull-down experiment using Snf21 fused to a protein A tag. We then analysed the coprecipitation of PK epitope-tagged Ssl3, the fission yeast ortholog of the cohesin loader subunit Scc4. Ssl3 was retrieved together with Snf21 (Fig. 1c), but not from a control strain lacking the Snf21 protein A tag. This confirms that a physical interaction between the RSC chromatin remodeller and the cohesin loader is also a conserved feature, at least between the budding yeast and fission yeast species.

## Requirements for cohesin loading onto chromatin

**Fig. 2:**
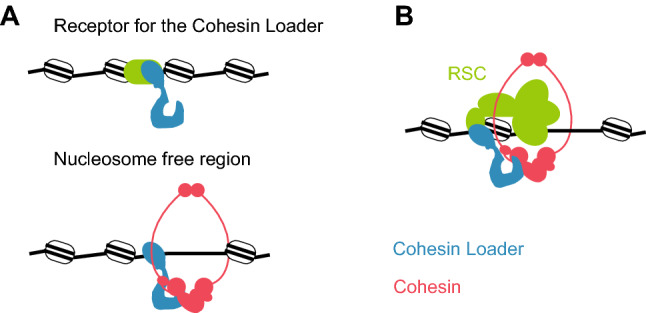
Cohesin loading onto chromatin **a** Cohesin loading onto chromatin requires a receptor for the cohesin loader and a nucleosome-free region. **b** RSC acts as chromatin receptor for the cohesin loader and concurrently produces nucleosome-free DNA for the cohesin loading reaction

The study of the role of RSC in cohesin loading onto chromosomes revealed that cohesin access to chromatin has two requisites. RSC serves both as a chromatin receptor for the cohesin loader and it provides nucleosome-free DNA for cohesin loading (Fig. 2a), (Muñoz et al. 2019). The requirement of a nucleosome-free stretch of DNA as a substrate for cohesin loading points to chromatin remodellers as ideal places for where cohesin loading can suitably take place. Due to their ability to alter nucleosome positioning, while simultaneously serving as anchors for the cohesin loader, a chromatin remodeller can combine both requirements (Fig. 2b). Out of the four chromatin remodeller categories, members of the SWI/SNF family have the unique ability to evict nucleosomes, thereby providing effective DNA access (Clapier et al. 2017). These considerations can rationalize why RSC appears to represent the principal cohesin loading receptor in both budding and fission yeast.

The Scc2–Scc4 cohesin loader can be functionally divided into two parts (Chao et al. 2015, 2017). Scc4 forms an alpha-helical assembly around the Scc2 N-terminus and promotes interactions with RSC, as well as with an additional centromeric chromatin receptor (Hinshaw et al. 2017; Muñoz et al. 2019). This module is essential for cohesin loading in vivo, but is dispensable for the ability of the cohesin loader to stimulate topological cohesin loading onto DNA in vitro. Using a naked DNA substrate in vitro, the C-terminal portion of both budding and fission yeast Scc2 (Scc2C) is sufficient to promote cohesin loading (Chao et al. 2015; Minamino et al. 2018). Artificially tethering budding yeast Scc2C–RSC, using the GFP and GFP-binding protein pair, circumvents the need for Scc4 and restores cohesin loading by this engineered cohesin loading module. This ability of an engineered RSC–Scc2C module to load cohesin opened the possibility to conduct a search for alternate cohesin loader receptors on chromatin. Apart from RSC, tethering Scc2C to the Isw1 or Chd1 chromatin remodellers, but not to many other chromatin components, also created functional in vivo cohesin loaders (Muñoz et al. 2019). This suggests that chromatin remodellers other than RSC can in principle substitute as cohesin loader receptors. ISWI and CHD1 family chromatin remodellers are mainly thought to act in assembling and regularly spacing nucleosomes (Clapier et al. 2017). How could these remodellers provide the DNA substrate for cohesin loading? It could be that their basal ability to move DNA along nucleosomes suffices to create a suitable substrate for cohesin loading. Alternatively, these engineered cohesin loading modules might depend on a window following DNA replication during S phase, when new chromatin assembly takes place, for the majority of their cohesin loading. We note that other replication-associated factors such as pre-replication complexes in *X. laevis* (Takahashi et al. 2008) or the MCM helicase in HeLa cells (Zheng et al. 2018) have been put forward as cohesin-loader receptors. Whether ISWI and CHD family remodellers promote cohesin loading also in a wild-type cell background will be important to explore. An initial analysis in human cells suggests that this might be the case (Hakimi et al. 2002).

Other possible chromatin receptors for the cohesin loader are the human mediator complex, that is found at active promoters (Kagey et al. 2010), the yeast kinetochore complex Ctf19 (Hinshaw et al. 2017) and the heterochromatin protein HP1γ at sites of DNA damage (Bot et al. 2017). Whether centromeric chromatin and heterochromatin hold distinct qualities that make them permissive for cohesin loading without assistance of chromatin remodellers, or whether chromatin remodellers are required cofactors for cohesin loading at these sites, remains to be determined. Nucleosomes at the centromere are marked by the presence the Histone 3 variant CENP-A. Recent structural studies suggest that these nucleosomes adopt an untwisted configuration (Takizawa et al. 2020; Yan et al. 2019), that might increase chromatin accessibility. At the same time, cohesin loading at budding yeast centromeres remains dependent on the RSC chromatin remodeller, despite the presence of the additional Ctf19 receptor (Muñoz et al. 2019). This emphasizes the importance of chromatin remodellers during cohesin loading onto chromosomes in vivo.

Apart from cohesin, all eukaryotes contain at least two other SMC complex family members, condensin and the Smc5–Smc6 complex (Uhlmann 2016). These SMC complexes do not make use of a specialised loading factor. Do they nevertheless require chromatin remodellers that provide nucleosome-free regions for their association to chromosomes? Budding yeast condensin has been reported to interact with transcription factors (Kim et al. 2016) and its chromosomal locations overlap with those of the cohesin loader at the promoters of highly transcribed genes (D'Ambrosio et al. 2008). Condensin also loads at open promoter regions in *C. elegans* and human cells (Kranz et al. 2013; Sutani et al. 2015). While the chromatin receptors may differ, the requirement for nucleosome-free DNA in open chromatin might be in common between cohesin and condensin. In fission yeast, the RSC complex has been found to be required for the condensin loading onto chromosomes, consistent with this notion (Toselli-Mollereau et al. 2016). Whether RSC engages in direct physical contact with condensin to facilitate its loading onto chromosomes is still to be determined. Nevertheless, the requirement of RSC for condensin binding to chromatin highlights the need of nucleosome-free DNA for SMC complexes beyond cohesin. Together, these findings indicate that the principles of cohesin loading onto chromatin are shared by other SMC complexes and emphasize the tight relationship between the local chromatin structure, determined by nucleosome positioning, and SMC-dependent higher order chromatin architecture.

## Methods

### Yeast strains and culture

Epitope tagging of endogenous genes and gene deletions were performed by gene targeting using polymerase chain reaction (PCR) products. The strains used in this study are listed in Table 1. Cells were asynchronously grown at 30 °C in YES broth. To deplete Snf21, *snf21-36* cells were cultured at 25 °C and when they reached an optical density OD_600_ = 0.2, they were shifted to 37 °C for the indicated times.

**Table 1:** List of *S. pombe* strains used in this study

Name	Genotype
Y4492	*h*^*−*^*, cen2::kanR-ura4*^*+*^*-lacO, his7*^*+*^*::GFP-LacI-NLS, leu1-32, lys1-131, ura4-D18, ade6-M210*
Y5834	*h*^*−*^*, cen2::kanR-ura4*^*+*^*-lacO, his7*^*+*^*::GFP-LacI-NLS, snf22::natMX, leu1-32, lys1-131, ura4-D18, ade6-M210*
Y5833	*h*^*−*^*, cen2::kanR-ura4*^*+*^*-lacO, his7*^*+*^*::GFP-LacI-NLS, hrp1::natMX, leu1-32, lys1-131, ura4-D18, ade6-M210*
Y5826	*h*^*−*^*, cen2::kanR-ura4*^*+*^*-lacO, his7*^*+*^*::GFP-LacI-NLS, hrp3::natMX, leu1-32, lys1-131, ura4-D18, ade6-M210*
Y5827	*h*^*−*^*, cen2::kanR-ura4*^*+*^*-lacO, his7*^*+*^*::GFP-LacI-NLS, mit1::natMX, leu1-32, lys1-131, ura4-D18, ade6-M210*
Y5828	*h*^*−*^*, cen2::kanR-ura4*^*+*^*-lacO, his7*^*+*^*::GFP-LacI-NLS, swr1::natMX, leu1-32, lys1-131, ura4-D18, ade6-M210*
Y5825	*h?, snf21-36, cen2::kanR-ura4*^*+*^*-lacO, his7*^*+*^*::GFP-LacI-NLS, leu1-32, lys1-131, ura4-D18, ade6-M210*
Y6000	*h*^*−*^*, ChrI::1.95 Mb-LacO-natMX6, his7*^*+*^*::LacI-GFP, lys1-131, ura4-D18, ade6-M210*
Y6069	*h*^*−*^*, ChrI::1.95 Mb-LacO-natMX6, his7*^*+*^*::LacI-GFP, snf22::KanMX, lys1-131, ura4-D18, ade6-M210*
Y6065	*h*^*−*^*, ChrI::1.95 Mb-LacO-natMX6, his7*^*+*^*::LacI-GFP, hrp1::KanMX, lys1-131, ura4-D18, ade6-M210*
Y6066	*h*^*−*^*, ChrI::1.95 Mb-LacO-natMX6, his7*^*+*^*::LacI-GFP, hrp3::KanMX, lys1-131, ura4-D18, ade6-M210*
Y6064	*h*^*−*^*, ChrI::1.95 Mb-LacO-natMX6, his7*^*+*^*::LacI-GFP, mit1::KanMX, lys1-131, ura4-D18, ade6-M210*
Y6067	*h*^*−*^*, ChrI::1.95 Mb-LacO-natMX6, his7*^*+*^*::LacI-GFP, swr1::KanMX, lys1-131, ura4-D18, ade6-M210*
Y6107	*h*^*−*^*, snf21-36, ChrI::1.95 Mb-LacO-natMX6, his7*^*+*^*::LacI-GFP, lys1-131, ura4-D18, ade6-M210*
Y5987	*h*^*−*^*, Ssl3-PK3::hphMX, leu1-32, ura4-D18*
Y5953	*h*^*−*^*, Snf21-PA::KanMX, leu1-32, ura4-D18*
Y5988	*h*^*−*^*, Ssl3-PK3::hphMX, Snf21-PA::kanMX, leu1-32, ura4-D18*

### Sister chromatid cohesion assay

Cells carrying the GFP-LacI repressor and an array of Lac operators either next to the centromere of chromosome II to measure cohesion at the centromere or at position 1.95 Mb of the chromosome I to measure cohesion of a chromosome arm were fixed with 70% ethanol. Images were acquired using a DeltaVision wide-field fluorescence microscope (GE Healthcare). z-stacks with 15 images at 0.1 μm intervals were acquired and merged by maximum intensity projection. Quantification of the percentage of cells showing two separated GFP foci was performed using Fiji.

### Coimmunprecipitation

Cell extracts were prepared in EBX buffer (50 mM HEPES–KOH pH 7.5, 100 mM KCl, 2.5 mM MgCl_2_, 10% glycerol, 0.25% Triton X-100, 1 mM DTT, protease inhibitors and benzonase) using glass bead breakage in a cooled Multi-Beads Shocker (Yasui Kikai). Extracts were cleared by centrifugation, precleared and incubated with IgG-coated Dynabeads (ThermoFisher) to adsorb protein A (PA)-tagged Snf21. Beads were extensively washed and elution was carried out in SDS-PAGE loading buffer. Whole cell extracts and precipitates were separated by SDS–polyacrylamide gel electrophoresis before transfer to nitrocellulose membranes. Ssl3-PK was detected using the mouse monoclonal anti-V5 clone MCA1360 antibody (Biorad) and Snf21-PA with a rabbit peroxidase anti-peroxidase antibody (Sigma-Aldrich).

## References

[CR1] Bot C, Pfeiffer A, Giordano F, Manjeera DE, Dantuma NP, Strom L (2017). Independent mechanisms recruit the cohesin loader protein NIPBL to sites of DNA damage. J Cell Sci.

[CR2] Chao WC, Murayama Y, Munoz S, Costa A, Uhlmann F, Singleton MR (2015). Structural studies reveal the functional modularity of the Scc2-Scc4 cohesin loader. Cell Rep.

[CR3] Chao WC, Murayama Y, Munoz S, Jones AW, Wade BO, Purkiss AG, Hu XW, Borg A, Snijders AP, Uhlmann F, Singleton MR (2017). Structure of the cohesin loader Scc2. Nat Commun.

[CR4] Ciosk R, Shirayama M, Shevchenko A, Tanaka T, Toth A, Shevchenko A, Nasmyth K (2000). Cohesin's binding to chromosomes depends on a separate complex consisting of Scc2 and Scc4 proteins. Mol Cell.

[CR5] Clapier CR, Iwasa J, Cairns BR, Peterson CL (2017). Mechanisms of action and regulation of ATP-dependent chromatin-remodelling complexes. Nat Rev Mol Cell Biol.

[CR6] D'Ambrosio C, Schmidt CK, Katou Y, Kelly G, Itoh T, Shirahige K, Uhlmann F (2008). Identification of cis-acting sites for condensin loading onto budding yeast chromosomes. Genes Dev.

[CR7] Fryns JP (1986). On the nosology of the Cornelia de Lange and Coffin-Siris syndromes. Clin Genet.

[CR8] Garcia MA, Koonrugsa N, Toda T (2002). Spindle-kinetochore attachment requires the combined action of Kin I-like Klp5/6 and Alp14/Dis1-MAPs in fission yeast. EMBO J.

[CR9] Gillespie PJ, Hirano T (2004). Scc2 couples replication licensing to sister chromatid cohesion in *Xenopus* egg extracts. Curr Biol.

[CR10] Hakimi MA, Bochar DA, Schmiesing JA, Dong Y, Barak OG, Speicher DW, Yokomori K, Shiekhattar R (2002). A chromatin remodelling complex that loads cohesin onto human chromosomes. Nature.

[CR11] Hinshaw SM, Makrantoni V, Harrison SC, Marston AL (2017). The kinetochore receptor for the cohesin loading complex. Cell.

[CR12] Huang J, Hsu JM, Laurent BC (2004). The RSC nucleosome-remodeling complex is required for Cohesin's association with chromosome arms. Mol Cell.

[CR13] Kagey MH, Newman JJ, Bilodeau S, Zhan Y, Orlando DA, van Berkum NL, Ebmeier CC, Goossens J, Rahl PB, Levine SS, Taatjes DJ, Dekker J, Young RA (2010). Mediator and cohesin connect gene expression and chromatin architecture. Nature.

[CR14] Kim KD, Tanizawa H, Iwasaki O, Noma K (2016). Transcription factors mediate condensin recruitment and global chromosomal organization in fission yeast. Nat Genet.

[CR15] Krantz ID, McCallum J, DeScipio C, Kaur M, Gillis LA, Yaeger D, Jukofsky L, Wasserman N, Bottani A, Morris CA, Nowaczyk MJ, Toriello H, Bamshad MJ, Carey JC, Rappaport E, Kawauchi S, Lander AD, Calof AL, Li HH, Devoto M, Jackson LG (2004). Cornelia de Lange syndrome is caused by mutations in NIPBL, the human homolog of Drosophila melanogaster Nipped-B. Nat Genet.

[CR16] Kranz AL, Jiao CY, Winterkorn LH, Albritton SE, Kramer M, Ercan S (2013). Genome-wide analysis of condensin binding in Caenorhabditis elegans. Genome Biol.

[CR17] Litwin I, Wysocki R (2018). New insights into cohesin loading. Curr Genet.

[CR18] Lopez-Serra L, Kelly G, Patel H, Stewart A, Uhlmann F (2014). The Scc2-Scc4 complex acts in sister chromatid cohesion and transcriptional regulation by maintaining nucleosome-free regions. Nat Genet.

[CR19] Minamino M, Higashi TL, Bouchoux C, Uhlmann F (2018). Topological in vitro loading of the budding yeast cohesin ring onto DNA. Life Sci Alliance.

[CR20] Muñoz S, Minamino M, Casas-Delucchi CS, Patel H, Uhlmann F (2019). A Role for chromatin remodeling in Cohesin loading onto chromosomes. Mol Cell.

[CR21] Murayama Y, Uhlmann F (2014). Biochemical reconstitution of topological DNA binding by the cohesin ring. Nature.

[CR22] Nasmyth K, Haering CH (2009). Cohesin: its roles and mechanisms. Annu Rev Genet.

[CR23] Petela NJ, Gligoris TG, Metson J, Lee BG, Voulgaris M, Hu B, Kikuchi S, Chapard C, Chen W, Rajendra E, Srinivisan M, Yu H, Lowe J, Nasmyth KA (2018). Scc2 Is a potent activator of Cohesin's ATPase that promotes loading by binding Scc1 without Pds5. Mol Cell.

[CR24] Peters JM, Nishiyama T (2012). Sister chromatid cohesion. Cold Spring Harb Perspect Biol.

[CR25] Petrova B, Dehler S, Kruitwagen T, Heriche JK, Miura K, Haering CH (2013). Quantitative analysis of chromosome condensation in fission yeast. Mol Cell Biol.

[CR26] Santen GW, Aten E, Sun Y, Almomani R, Gilissen C, Nielsen M, Kant SG, Snoeck IN, Peeters EA, Hilhorst-Hofstee Y, Wessels MW, den Hollander NS, Ruivenkamp CA, van Ommen GJ, Breuning MH, den Dunnen JT, van Haeringen A, Kriek M (2012). Mutations in SWI/SNF chromatin remodeling complex gene ARID1B cause Coffin-Siris syndrome. Nat Genet.

[CR27] Sutani T, Sakata T, Nakato R, Masuda K, Ishibashi M, Yamashita D, Suzuki Y, Hirano T, Bando M, Shirahige K (2015). Condensin targets and reduces unwound DNA structures associated with transcription in mitotic chromosome condensation. Nat Commun.

[CR28] Takahashi TS, Basu A, Bermudez V, Hurwitz J, Walter JC (2008). Cdc7-Drf1 kinase links chromosome cohesion to the initiation of DNA replication in *Xenopus* egg extracts. Genes Dev.

[CR29] Takizawa Y, Ho CH, Tachiwana H, Matsunami H, Kobayashi W, Suzuki M, Arimura Y, Hori T, Fukagawa T, Ohi MD, Wolf M, Kurumizaka H (2020). Cryo-EM structures of centromeric tri-nucleosomes containing a central CENP-A nucleosome. Structure.

[CR30] Toselli-Mollereau E, Robellet X, Fauque L, Lemaire S, Schiklenk C, Klein C, Hocquet C, Legros P, N'Guyen L, Mouillard L, Chautard E, Auboeuf D, Haering CH, Bernard P (2016). Nucleosome eviction in mitosis assists condensin loading and chromosome condensation. EMBO J.

[CR31] Tsurusaki Y, Okamoto N, Ohashi H, Mizuno S, Matsumoto N, Makita Y, Fukuda M, Isidor B, Perrier J, Aggarwal S, Dalal AB, Al-Kindy A, Liebelt J, Mowat D, Nakashima M, Saitsu H, Miyake N, Matsumoto N (2014). Coffin-Siris syndrome is a SWI/SNF complex disorder. Clin Genet.

[CR32] Uhlmann F (2016). SMC complexes: from DNA to chromosomes. Nat Rev Mol Cell Biol.

[CR33] Villa-Hernandez S, Bermejo R (2018). Cohesin dynamic association to chromatin and interfacing with replication forks in genome integrity maintenance. Curr Genet.

[CR34] Yague-Sanz C, Vazquez E, Sanchez M, Antequera F, Hermand D (2017). A conserved role of the RSC chromatin remodeler in the establishment of nucleosome-depleted regions. Curr Genet.

[CR35] Yamada K, Hirota K, Mizuno K, Shibata T, Ohta K (2008). Essential roles of Snf21, a Swi2/Snf2 family chromatin remodeler, in fission yeast mitosis. Genes Genet Syst.

[CR36] Yan K, Yang J, Zhang Z, McLaughlin SH, Chang L, Fasci D, Ehrenhofer-Murray AE, Heck AJR, Barford D (2019). Structure of the inner kinetochore CCAN complex assembled onto a centromeric nucleosome. Nature.

[CR37] Zheng G, Kanchwala M, Xing C, Yu H (2018). MCM2-7-dependent cohesin loading during S phase promotes sister-chromatid cohesion. Elife.

[CR38] Zuin J, Franke V, van Ijcken WF, van der Sloot A, Krantz ID, van der Reijden MI, Nakato R, Lenhard B, Wendt KS (2014). A cohesin-independent role for NIPBL at promoters provides insights in CdLS. PLoS Genet.

